# Enhancer of Zeste Homolog 2 as an Independent Prognostic Marker for Cancer: A Meta-Analysis

**DOI:** 10.1371/journal.pone.0125480

**Published:** 2015-05-14

**Authors:** Shuling Chen, Lixia Huang, Kaiyu Sun, Dexi Wu, Minrui Li, Manying Li, Bihui Zhong, Minhu Chen, Shenghong Zhang

**Affiliations:** 1 Division of Gastroenterology, The First Affiliated Hospital, Sun Yat-sen University, Guangzhou, P.R. China; 2 Division of Respiration, The First Affiliated Hospital, Sun Yat-sen University, Guangzhou, P.R. China; 3 Division of Gastrointestinal Surgery, The First Affiliated Hospital, Sun Yat-sen University, Guangzhou, P.R. China; 4 Division of Cardiology, The First Affiliated Hospital, Sun Yat-sen University, Guangzhou, P.R. China; Ospedale Pediatrico Bambino Gesu', ITALY

## Abstract

**Background:**

Novel biomarkers are of particular interest for predicting cancer prognosis. This study aimed to explore the associations between enhancer of zeste homolog 2 (EZH2) and patient survival in various cancers.

**Methods:**

Relevant literature was retrieved from PubMed and Web of Science databases. Pooled hazard ratios (HRs), odds ratios (ORs), and 95% confidence intervals (CIs) were calculated.

**Results:**

Forty-nine studies (8,050 patients) were included. High EZH2 expression was significantly associated with shorter overall (hazard ratio [HR] 1.74, 95% CI: 1.46–2.07), disease-free (HR 1.59, 95% CI: 1.27–1.99), metastasis-free (HR 2.19, 95% CI: 1.38–3.47), progression-free (HR 2.53, 95% CI: 1.52–4.21), cancer-specific (HR 3.13, 95% CI: 1.70–5.74), and disease-specific (HR 2.29, 95% CI: 1.56–3.35) survival, but not recurrence-free survival (HR 1.38, 95% CI: 0.93–2.06). Moreover, EZH2 expression significantly correlated with distant metastasis (OR 3.25, 95% CI: 1.07–9.87) in esophageal carcinoma; differentiation (OR 3.00, 95% CI: 1.37–6.55) in non-small cell lung cancer; TNM stage (OR 3.18, 95% CI: 2.49–4.08) in renal cell carcinoma; and histological grade (OR 4.50, 95% CI: 3.33–6.09), estrogen receptor status (OR 0.15, 95% CI: 0.11–0.20) and progesterone receptor status (OR 0.30, 95% CI: 0.23–0.39) in breast cancer.

**Conclusions:**

Our results suggested that EZH2 might be an independent prognostic factor for multiple survival measures in different cancers.

## Introduction

Cancer has become a major cause of morbidity and mortality worldwide, in both developed countries and developing areas [1]. The enduring battle against this disease has yet to successfully offer its patients a favorable long-term prognosis. Early detection and treatment remains the best strategy for improving patients’ quality of life and prognosis. Although many studies have attempted to develop tools and identify biomarkers for the early diagnosis or prognostic prediction of various human cancers, their successes have been limited. Therefore, novel effective biomarkers remain a topic of special interest in the field.

Enhancer of zeste homologue 2 (EZH2), a core protein of the polycomb-repressive complex 2 (PRC2), plays a vital role in the epigenetic maintenance of histone H3 lysine 27 (H3K27) repressive chromatin mark. Emerging data have demonstrated that EZH2 is aberrantly expressed in various types of human cancers, including breast, brain, colon, gastric, liver, and lung cancers. *In vitro* overexpression of EZH2 in cancer cell lines has been shown to activate their proliferation, migration, and invasion abilities.^2^ In contrast, knockdown of EZH2 using siRNA or shRNA results in cell growth inhibition and suppression of oncogenic capacity[2]. The mechanisms underlying EZH2 regulation in cancer are unclear. EZH2 is thought to work in association with other PRC2 components and subsequently catalyze H3K27 or interact with DNA methyltransferases. It has been reported to be involved in many cellular processes, including the cell cycle, differentiation, senescence, and cancer [2–4]. Numerous reports have suggested that EZH2 expression might play meaningful prognostic roles in certain cancers. However, most studies examining the implication of EZH2 expression are limited by their small sample sizes. Therefore, we conducted a systematic review and quantitative meta-analysis to clarify the prognostic value of EZH2 expression in human cancers.

## Materials and Methods

### Study strategy

The present study was performed according to recent guidelines for meta-analyses and systematic reviews of tumor marker prognostic studies [5,6]. To obtain appropriate materials for this review, two authors (SL Chen and SH Zhang) independently searched PubMed and ISI Web of Science databases to identify all relevant articles about EZH2 as a prognostic factor for cancer patient survival. The literature search ended on May 8, 2014. Both Medical Subject Headings and free-text terms, such as “enhancer of zeste homologue 2,” “EZH2,” “polycomb repressive complex 2,” “PRC2,” “cancer,” “carcinoma,” “tumor,” “prognosis,” “prognostic,” and “survival,” were used to increase the search sensitivity.

### Study selection

Two investigators (SL Chen and SH Zhang) independently evaluated all eligible studies and extracted their data. Studies were considered eligible if they met the following criteria: human cancer was studied, excepting blood carcinomas; EZH2 expression was determined in human tissue using immunohistochemistry (IHC); the relationship between EZH2 expression and survival was examined; sufficient data were provided to estimate hazard ratios (HRs) for survival rates and their 95% confidence intervals (CIs). If data subsets were published in more than one article, only the most recent article was included. Citations were limited to those published in English. Animal studies and single case reports were excluded. If data could not be extracted or calculated from the original article, the study was also excluded. Disagreements were resolved through discussion with a third investigator (LX Huang).

### Data extraction

The two investigators (SL Chen and LX Huang) extracted data independently and reached a consensus on all items. Data on the following characteristics were collected from each research article: author, journal name, year of publication, country of the population enrolled, number of patients, elevated EZH2 expression, EZH2 detection methods, cut-off values, overall survival (OS), disease-free survival (DFS), metastasis-free survival (MFS), recurrence-free survival (RFS), cancer-specific survival (CSS), disease-specific survival (DSS), progression-free survival (PFS), and failure-free survival (FFS).

### Quality assessment of the primary studies

Quality was independently assessed by three investigators (SH Zhang, SL Chen, and MH Chen). All eligible studies were scored as previously reported [7,8]. Four main methods were evaluated: scientific design, laboratory methodology, generalizability, and analysis of results. There were four to seven items for each method. Each item was scored as follows: if it was clearly and accurately defined, two points; if it was unclear or incomplete, one point; and if it was not defined or inadequate, zero points. The final scores were expressed as percentages, with a higher percentage denoting better methodological quality (> 80%).

### Statistical analysis

HRs were extracted using three previously published methods [9,10]. The first option, which offered the highest accuracy, was to obtain such estimates directly from the original study or to calculate HRs from the O-E statistic and variance (if available). If such a method was not feasible, relevant data, such as the number of patients at risk in each group, the number of events, and the log-rank statistics or p-values, were used to calculate HRs. However, in some studies, HRs were only represented as Kaplan-Meier survival curves. We therefore had to approximate the HRs by extracting survival rates at specified time points from the provided survival curves, assuming that the rate of censored patients during follow-up was constant. Engauge Digitizer version 2.11 was used to obtain the necessary points on the curves, to minimize the potential bias of visual approximation.

Results were reported as pooled HRs or odd ratios (ORs) and their corresponding 95% CI. They were first estimated using the random-effect model to identify heterogeneity. If the heterogeneity was not significant, estimation using the fixed-effect model (Mantel-Haenszel) was performed [11]. The results reported in the following text are the values that were calculated from the corresponding appropriate analysis models.

Heterogeneity was assessed using the chi-square-based Q test, for which a p-value of <0.05 was considered statistically significant. The I^2^ statistic measures the degree of inconsistency among the studies, with larger values of I^2^ indicating increased heterogeneity. I^2^ values of 25%, 50%, and 75% reflect low, moderate, and high degrees of heterogeneity, respectively. A subgroup analysis was performed according to the following factors, when appropriate: region, sample size, type of carcinoma, treatment, and quality score. Univariate meta-regression was conducted to explore the potential heterogeneity in the analysis of the association between EZH2 and survival when the number of included studies reached ten. Furthermore, factors identified as significant by univariate analysis were further analyzed with multivariate meta-regression if necessary. We also performed a sensitivity analysis to test the contribution of some studies to the overall effect and the robustness of the pooled ORs/HRs. Sensitivity was assessed by serial omission of each study. Furthermore, cumulative meta-analyses were conducted to detect the dynamic trends of HRs for OS, DFS, and RFS over time.

Publication bias was qualitatively evaluated using graphical funnel plots, whereas the Begg’s rank correlation test and Egger’s regression asymmetry test were employed as quantitative indicators of the presence of publication bias [12,13]. Since Begg’s test does not provide robust results in small meta-analyses, we only performed Egger’s test for analyses that included less than 15 studies. Furthermore, we drew our conclusions regarding publication bias from the combined results of Egger’s test and the graphical presentation of the funnel plot. We selected this approach to evaluating publication bias because Begg’s test has limited sensitivity to assess asymmetry of the funnel plot when the number of included studies is less than 75. The statistical analysis was performed using Stata software version 12.0 (Stata, College Station, TX).

## Results

**Fig 1:**
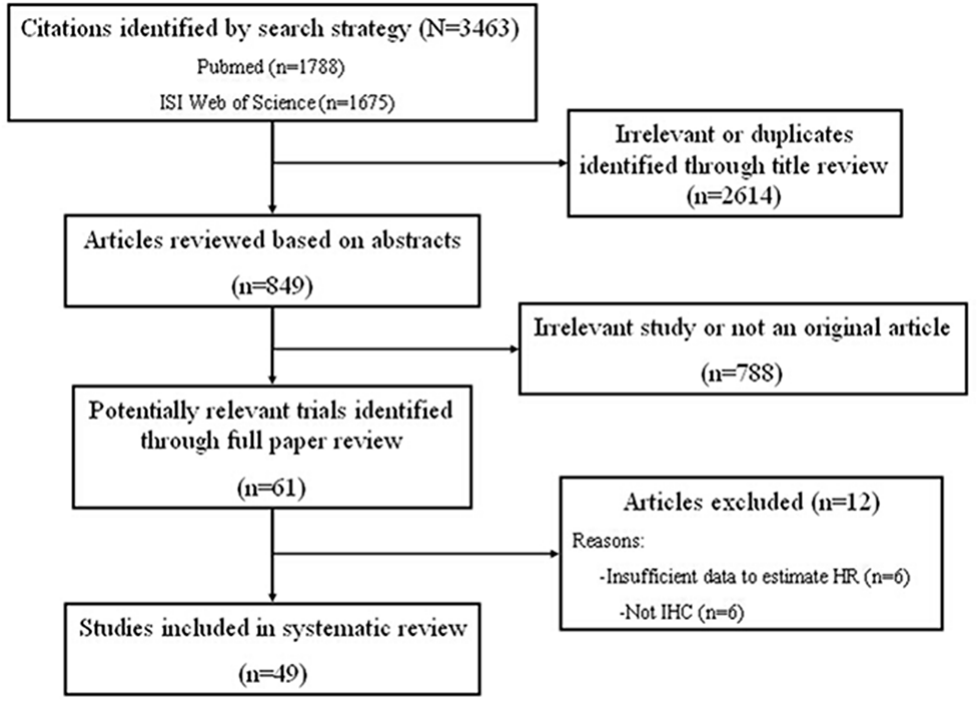
Schemata of the systematic review.

We identified 3,463 articles using the literature search strategy shown in **Fig 1**. Irrelevant or duplicate reports were excluded by reviewing titles and abstracts. The remaining articles were reviewed in full and excluded if EZH2 expression was not evaluated through IHC or if there were insufficient data to calculate or approximate HRs. Finally, 49 articles were included in the present study.

The detailed characteristics of all included studies are summarized in **S1 Table** [14–62]. Most of the studies were published within the past ten years (range, 2005–2014) whereas the accrual periods were between 1979 and 2012. We evaluated studies from 12 different countries, including 14 studies from China, 11 from Japan, nine from the United States, four from Korea, three from Norway, and the remaining nine from seven other countries. These eligible studies enrolled 8,050 participants in total, with minimum and maximum sample sizes of 21 and 696, respectively (mean, 164.3 patients).

Twenty-two different types of carcinoma were analyzed, most of which were carcinomas of the digestive system (four each of colorectal, esophageal, and gastric carcinoma; one hepatocellular carcinoma; one cholangiocarcinoma; and one gallbladder adenocarcinoma). Other cancer types were also investigated, including seven studies on breast cancer, six on renal cell carcinoma, four on non-small cell lung cancer (NSCLC), three on prostate cancer, three on oral tongue squamous carcinoma, and the remaining 14 studies on 11 different types of cancer.

Treatment information was not available in three studies, and of the remaining 46, only 43 enrolled patients underwent surgery. Outcome measures were clearly defined in 18 studies, and multivariate analyses were performed in 26 studies (53.1%). OS, DFS, RFS, MFS, PFS, CSS, DSS, and FFS were the main outcome measures in the included studies. We decided to focus on OS, DFS, and RFS. More than half of the included studies (30/49, 61.2%) achieved a quality score of ≥80%.

A total of 68 HRs were extracted from 49 studies, including 40 for OS; ten for DFS; six for RFS; three each for MFS, CSS/CRS, and DSS; two for PFS and one for FFS. Of these, 40 HRs were directly obtained and nine were approximated from the total number of events and the log-rank statistics or p-values. The remaining 19 were estimated from reconstruct survival curves. High or positive EZH2 expression was identified as an indicator of poor OS (29/40, 72.5%), DFS (7/10, 70.0%), RFS (3/7, 42.9%), MFS (2/3, 66.7%), DSS (3/3, 100%), PFS (2/2, 100%), and CSS (3/3, 100%).

### The prognostic significance of high EZH2 expression in OS in multiple cancers

**Fig 2:**
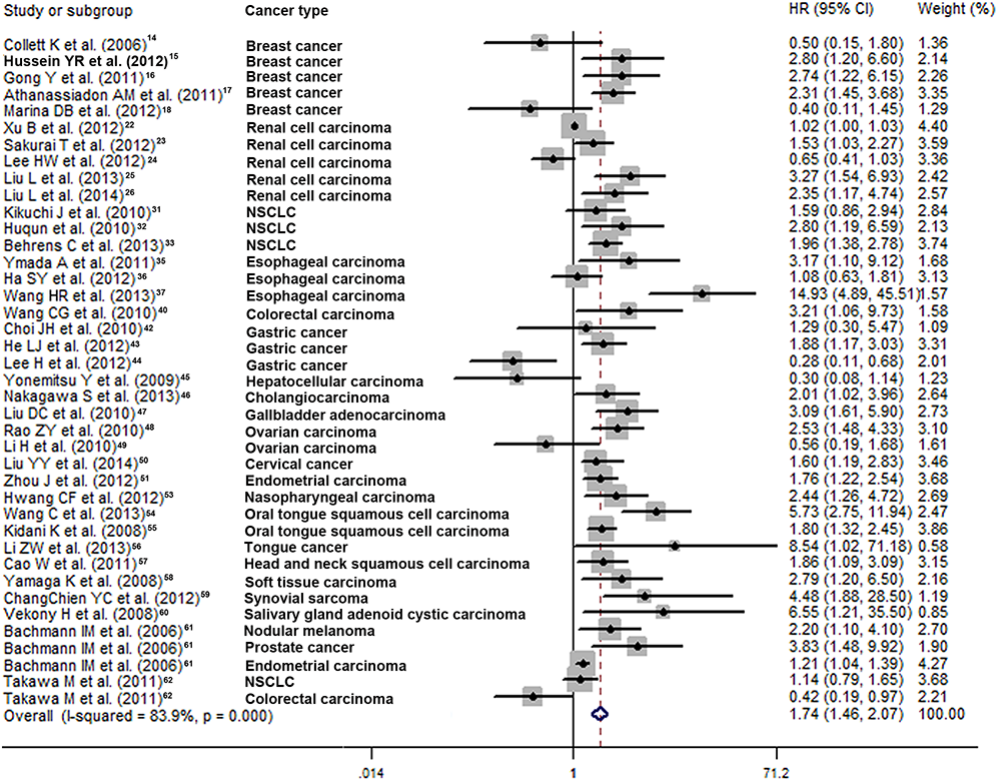
Forest plot for the meta-analysis of the association between EZH2 expression and overall survival in various cancer types. The segments represent the 95% confidence intervals (CIs) of each study. The diamond represents the overall effect size, and the diamond’s width represents the overall 95% CI.

**Fig 3:**
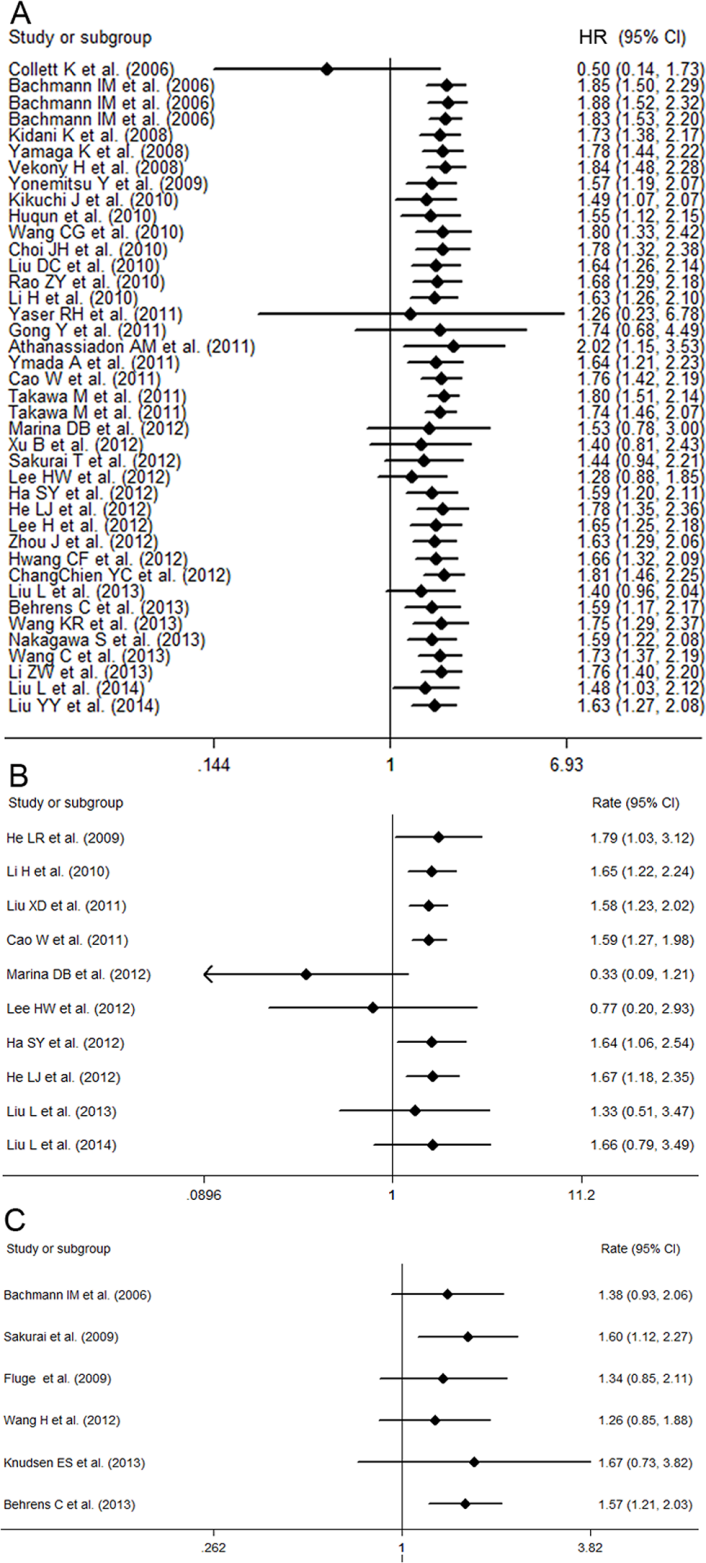
Forest plots for the accumulative meta-analyses of the association between EZH2 expression and cancer survival. The following cancer survival measures were analyzed: OS (A), DFS (B), and RFS (C). The segments represent the 95% confidence intervals (CIs) of each study. The diamond represents the overall effect size, and the diamond’s width represents the overall 95% CI.

The association between EZH2 expression and OS was reported in 40 studies enrolling 5,737 patients with various cancer types[14–18,22–26,31–33,35–37,40,43–51,53–62]. A combined analysis showed that high EZH2 expression predicted poor OS in cancer (HR 1.74, 95% CI: 1.46–2.07; *p*<0.00001) with significant heterogeneity (I^2^ = 83.9%) **(Table 1 and Fig 2)**. The cumulative meta-analysis showed that, with the exception of studies by Collett et al. (2006) [14], HRs tended to be rather stable over time, and the pooled HR (1.63, 95% CI: 1.27–2.08; *p*<0.00001) was similar to that found in the ordinary meta-analysis **(Fig 3A)**. Multivariate analysis was performed in 17 studies, and the pooled HR was 1.99 (95% CI: 1.51–2.62; *p*<0.00001) with pronounced heterogeneity (I^2^ = 64.4%) (**Figure A in S1 File**). Such results indicated that high EZH2 expression was an independent factor of poor OS in multiple cancers.

**Table 1:** Results of subgroup analysis of the association between nuclear EZH2 expression and OS of multiple cancers.

Subgroup analysis	No. of studies	No. of patients	Pooled HR	*p*-value	Meta regression (p-value)	Heterogeneity	
						I^2^	p-value (χ^2^)
**Overall survival**	40	5737	1.74 [1.46–2.07]	<0.00001	—	83.9%	0.000
**Region**					0.293		
Asian countries	23	3379	1.96 [1.50–2.56]	<0.00001		74.1%	0.000
Western countries	17	2258	1.52 [1.21–1.90]	<0.00001		82.2%	0.000
**Sample size**					0.009		
<150	21	2319	2.39 [1.77–3.24]	<0.00001		86.9%	0.000
≥150	19	3418	1.30 [1.02–1.67]	0.036		73.4%	0.000
**Type of cancer**					0.073		
Breast cancer	5	765	1.53 [0.78–3.00]	0.216		67.4%	0.015
Renal cell carcinoma	5	879	1.37 [0.91–2.07]	0.137		82.1%	0.000
NSCLC	3	580	1.65 [1.16–2.35]	0.006		51.3%	0.104
Digestive system carcinoma	10	1192	1.51 [0.85–2.69]	0.244		82.1%	0.000
Esophageal carcinoma	3	402	3.50 [0.74–16.54]	0.113		89.1%	0.000
Colorectal carcinoma	2	291	1.13 [0.16–8.21]	0.907		87.9%	0.004
Gastric cancer	3	432	0.88 [0.23–3.34]	0.854		85.3%	0.001
Female reproductive system carcinoma	4	831	1.77 [1.29–2.41]#	0.060		39.5%	0.158
Ovarian carcinoma	2	313	1.29 [0.30–5.59]	0.737		83.1%	0.015
Endometrial carcinoma	2	518	1.40 [0.98–2.01]	0.063		70.9%	0.064
Oral tongue carcinoma	3	234	3.59 [1.29–9.97]	0.014		79.4%	0.008
Treatment*	23	3542	2.13[1.62–2.80]	<0.00001	0.499	71.7%	0.000
Surgery without preoperative treatment	15	2443	2.31 [1.63–3.30]	<0.00001		74.6%	0.000
Surgery with preoperative treatment	8	1099	1.85 [1.14–2.98]	<0.00001		68.9%	0.002
**Quality score (%)**					0.603		
<83.0	21	2613	1.65 [1.25–2.19]	<0.00001		85.3%	0.000
≥83.0	19	3124	1.84 [1.45–2.33]	<0.00001		70.1%	0.000

*: Data regarding treatment were unclear in 16 studies. Further, the treatment in the study of nasopharyngeal carcinoma was radiotherapy, rather than surgery. Accordingly, a subgroup analysis of the treatment was performed after excluding these studies.

#: This result was estimated from the fixed-effect model.

OS: overall survival; HR: hazard ratio; NSCLC: non-small cell lung cancer.

Subgroup analysis revealed high EZH2 expression was significantly associated with poor OS in NSCLC (HR 1.65, 95% CI: 1.16–2.35; *p* = 0.006), female reproductive system carcinoma (HR 1.77, 95% CI: 1.29–2.41; *p* = 0.060), and oral tongue cancer (HR 3.59, 95% CI: 1.29–9.97; *p* = 0.014) **(Table 1)**. Unfortunately, we could not combine data from the other ten cancer types since only one study was included in each subgroup. The pooled HR was higher in studies of Asian patients than in studies of Western patients (HR 1.96, 95% CI: 1.50–2.56; *p*<0.00001). Further, pooled HRs were significantly greater for studies with smaller sample sizes (<150 vs. ≥150: HR 2.39, 95% CI: 1.77–3.24; *p*<0.00001) and those of patients undergoing surgery without preoperative treatment (no preoperative vs. preoperative therapy: HR 2.31, 95% CI: 1.63–3.30; *p*<0.00001). The results of studies with poor quality were similar to those of better quality (HR 1.65, 95% CI: 1.25–2.19; *p*<0.00001) **(Table 1)**.

Meta-regression analysis revealed that sample size might be a significant contributor to heterogeneity (*p* = 0.009), whereas publication year, region, type of cancer, treatment, and quality score were not (*p* = 0.073–0.925) **(Table 1)**.

Patients in the study conducted by Laitinen et al. (2008) received radiotherapy rather than surgery [30]. Moreover, 16 studies enrolled patients undergoing surgery but did not provide a clear description of treatments other than surgery[14,18,22–24,31–33,38,43,45,47,49,50,55,61]. Thus, a sensitivity analysis was performed in which we excluded these studies, and the HR increased slightly to 2.13 (95% CI: 1.62–2.80; *p*<0.00001). Significant heterogeneity was also detected (I^2^ = 71.7%). A further sensitivity analysis was performed with the study by Wang et al. excluded, because its HR was quite high relative to the other studies [55]. The HR dropped slightly to 1.72 (95% CI: 1.45–2.05; *p*<0.00001) after this exclusion.

There was evidence of publication bias in the meta-analysis of the association between EZH2 and OS, as indicated by Egger’s test (*p*<0.0001) and the relatively asymmetrical appearance of the funnel plot, even though Begg’s test was not significant (*p* = 0.159) **(S2 Table and S3 File)**. Furthermore, significant evidence of publication bias was observed in the following subgroups: publication year >2010 (*p* = 0.00), western countries (*p* = 0.01), sample size <150 (*p* = 0.00), quality score <83.0 (*p* = 0.01), quality score ≥ 83.0 (*p* = 0.02) and surgery without preoperative treatment (*p* = 0.04) **(S2 Table)**.

### The prognostic significance of high EZH2 expression in DFS, RFS, MFS, PFS, CSS, and DSS of cancer patients

Associations between high EZH2 expression and DFS, RFS, MFS, PFS, CSS, and DSS are presented in Tables 2 and 3 [18–20,21,24–28,30,33,34,36,39,41,43,44,52,53,57,62]. High EZH2 expression was significantly correlated with poor DFS **(Figure A in S2 File)**, MFS, PFS, CSS, and DSS, but not poor RFS **(Figure B in S2 File)**. Significant heterogeneity was observed for DFS and RFS **(Table 2)**, but not MFS, CSS, or DSS **(Table 3)**. Cumulative meta-analysis of DFS revealed that HRs in the studies by Marina et al. (2012) or Lee et al. (2012) deviated from the relatively stable trend of HRs over time**(Fig 3B)** [18,24]. However, for RFS, all the HRs were quite stable **(Fig 3C)**.

**Table 2:** Results of subgroup analysis of the association between nuclear EZH2 expression and DFS and RFS of multiple cancers.

Subgroup analysis	No. of studies	No. of patients	Pooled HR		*p*-value	Meta regression (p-value)	Heterogeneity	
			Fixed	Random			I^2^	p-value (χ^2^)
**Disease-free survival**	10	1531	1.42 [1.24–1.63]	1.59 [1.27–1.99]	<0.00001	—	53.4%	0.023
**Region**						0.313		
Asian countries	8	1257	1.52 [1.32–1.74]	1.64 [1.32–2.04]	<0.00001		50.4%	0.049
Western countries	2	274	1.13 [0.63–2.03]	0.79 [0.18–3.54]	0.762		76.6%	0.039
**Sample size**						0.737		
<120	4	379	1.57 [1.30–1.90]	1.60 [1.29–1.98]	<0.00001		16.0%	0.312
≥120	6	1152	1.42 [1.17–1.71]	1.54 [1.01–2.35]	0.046		67.0%	0.010
**Type of cancer**						0.943		
Renal cell carcinoma	3	714	1.54 [1.23–1.91]	2.25 [1.10–4.61]	0.027		77.4%	0.012
Esophageal carcinoma	2	262	1.49 [1.03–2.15]	1.54 [0.80–2.97]	0.197		68.2%	0.076
**Quality score (%)**						0.928		
<80.0	5	826	1.46 [1.25–1.70]	1.51 [1.23–1.86]	<0.00001		33.4%	0.199
≥80.0	5	705	1.61 [1.23–2.11]	1.57 [0.93–2.65]	0.093		69.1%	0.012
**Recurrence-free survival**	6	1131	1.46 [1.16–1.85]	1.38 [0.93–2.06]	0.113	—	54.9%	0.049
**Publication year**						—		
<2010	3	394	1.53 [1.07–2.18]	1.22 [0.39–3.80]	0.729		78.4%	0.010
≥2010	3	737	1.41 [1.04–1.92]	1.41 [1.04–1.92]	0.028		0.0%	0.419
**Sample size**						—		
<200	3	285	1.52 [1.10–2.10]	1.56 [0.89–2.73]	0.123		53.1%	0.119
≥200	3	846	1.40 [1.01–1.96]	1.06 [0.45–2.49]	0.889		70.2%	0.035
**Quality score (%)**						—		
≤84.5	4	737	1.47 [1.16–1.88]	1.47 [1.16–1.88]	0.002		0.0%	0.586
>84.5	2	394	1.33 [0.58–3.05]	0.92 [0.07–12.93]	0.954		89.0%	0.003

DFS: disease-free survival; RFS: recurrence-free survival; HR: hazard ratio.

**Table 3:** Results of the meta-analysis of the association between nuclear EZH2 expression and MFS, PFS, CSS and DSS of multiple cancers.

Meta-analysis	No. of studies	Cancer type	No. of patients	Pooled HR		*p-*value	Heterogeneity	
				Fixed	Random		I^2^	p-value (χ^2^)
**Metastasis-free survival**	3	Breast cancer; upper urinary tract carcinoma; esophageal carcinoma	749	2.19 [1.38–3.47]	2.22 [1.36–3.62]	0.0009	8.7%	0.334
**Progression-free survival**	2	Prostate cancer; esophageal carcinoma; salivary gland adenoid cystic carcinoma	311	2.64 [1.96–3.56]	2.53 [1.52–4.21]	0.0001	63.5%	0.098
**Cancer specific survival**	3	Breast cancer; upper urinary tract carcinoma; colorectal carcinoma	774	3.13 [1.70–5.74]	3.33 [1.45–7.64]	0.001	32.5%	0.227
**Disease specific survival**	3	Breast cancer; renal cell carcinoma; nasopharyngeal carcinoma	287	2.29 [1.56–3.35]	2.29 [1.56–3.35]	0.00006	0.0%	0.976

MFS: metastasis-free survival; PFS: progression-free survival; CSS: cancer specific survival; DSS: disease specific survival; HR: hazard ratio.

The pooled HRs of DFS and RFS were 2.00 (95% CI: 1.34–2.98; *p*<0.00001) and 1.61 (95% CI: 0.79–3.27; *p* = 0.113) in the combined analysis of five and three studies with multivariate analysis, respectively **(Figure B and C in S1 File)**. Significant heterogeneity was observed with DFS (I^2^ = 54.9%) but not RFS (I^2^ = 53.1%). Furthermore, since the above-mentioned results for MFS, CSS, and DSS were also obtained from multivariate analysis, high EZH2 expression might be an independent prognostic factor for DFS, MFS, CSS, and DSS in various cancer types.

The subgroup analysis according to sample size did not change the significant association between high EZH2 expression and DFS of cancer patients. The predictive value of EZH2 for DFS was significant for all subgroups except studies of Western patients, studies of esophageal carcinoma, and studies of quality scores ≥80.0 **(Table 2)**. On the other hand, the association between EZH2 and RFS was only significant in the subgroup of newer studies and studies with low quality **(Table 2)**. We did not perform subgroup analysis for MFS, PFS, CSS, or DSS, owing to the limited number of studies (two to three) on these outcome measures.

Similarly, meta-regression analysis was only performed for DFS **(Table 2)**, demonstrating that publication bias, region, sample size, and quality score did not significantly contribute to the bias among studies.

In the sensitivity analysis of DFS, the reports by He et al. (2010) and Liu et al. (2011) were excluded as patients in the former study were treated with radiotherapy rather than surgery[34,52], and as the latter study had a considerably smaller sample size and a considerably lower quality score than the overall trend. However, the exclusion of these two reports did not significantly alter the results (HR 1.54, 95% CI: 1.22–1.94; *p*<0.00001 and HR 1.66, 95% CI: 1.27–2.16; *p*<0.00001, respectively).

No evidence of publication bias was detected in for either DFS or RFS (DFS, *p* = 0.44 via Egger’s test; RFS, *p* = 0.48 via Egger’s test) **(S2 Table)**.

### The association between high EZH2 expression and clinicopathological characteristics

Nineteen studies provided sufficient data for the meta-analysis of the correlation between EZH2 expression and clinicopathological characteristics **(Table 4)** [14–17,19,21,23,24,26,31,32,34–37,41–44]. Esophageal carcinoma, renal cell carcinoma, breast cancer gastric cancer, and NSCLC were investigated in detail. For each disease, high EZH2 expression was significantly associated with some clinicopathological characteristics that are indicative of poor prognosis and disease aggressiveness. However, there was no immediately obvious pattern to these associations.

**Table 4:** Results of the meta-analysis of increased EZH2 expression and clinicopathological features of four types of cancer.

Clinicopathological features	No. of studies	No. of patients	Pooled OR		p-value	Heterogeneity	
			Fixed	Random		I^2^	p-value (χ^2^)
**Esophageal carcinoma**							
Distant metastasis	3	398	—	3.25 [1.07–9.87]	0.037	66.3%	0.052
Lymph node status	4	500	—	1.77 [0.88–3.56]	0.109	58.5%	0.065
T status	2	300	—	2.35 [0.83–6.67]	0.109	50.9%	0.153
**Renal cell carcinoma**							
pT stage	4	977	2.81 [2.21–3.57]	—	0.000	37.4%	0.187
Distant metastasis	4	977	—	2.06 [0.97–4.39]	0.061	63.4%	0.042
N status	2	620	5.68 [3.76–8.57]	—	0.000	0.00%	0.722
Fuhrman grade	4	977	—	2.51 [1.26–4.99]	0.009	81.0%	0.001
TNM stage	3	877	3.18 [2.49–4.08]	—	0.000	0.00%	0.416
**Breast cancer**							
Histological grade	5	1105	4.50 [3.33–6.09]	—	0.000	41.8%	0.143
N status	5	1105	1.45 [1.08–1.96]	1.60 [0.69–3.74]	0.276	82.8%	0.000
ER	5	1105	0.15 [0.11–0.20]	—	0.000	44.7%	0.124
PR	5	1105	0.30 [0.23–0.39]	—	0.000	15.7%	0.314
Her-2	4	915	—	2.08 [0.80–5.39]	0.131	76.0%	0.006
**Gastric cancer**							
pN stage	4	515	2.71 [1.70–4.34]	—	0.000	22.4%	0.276
Clinical stage	2	200	2.99 [1.59–5.62]	—	0.001	0.00%	0.810
T status	4	515	1.76 [1.10–2.80]	—	0.036	17.9%	0.301
Distant metastasis	2	254	2.65 [0.97–7.23]	—	0.058	16.9%	0.273
Lymphatic invasion	2	220	—	2.88 [0.98–8.49]	0.055	52.2%	0.148
Venous invasion	2	220	—	2.11 [0.56–7.89]	0.269	74.6%	0.047
**Non-small cell lung cancer**							
Differentiation	2	260	3.00 [1.37–6.55]	—	0.007	0.00%	0.345

OR: odds ratio; ER: estrogen receptor; PR: progesterone receptor; Her-2: human epidermal growth factor receptor 2.

Regarding predictive factors for breast cancer, high EZH2 expression was significantly associated with the absence of estrogen receptor (positive vs. negative: OR 0.15, 95% CI: 0.11–0.20) and progesterone receptor (positive vs. negative: OR 0.30, 95% CI: 0.23–0.39) expression.

## Discussion

This study aimed to assess the prognostic significance of EZH2 expression in cancer survival by exploring the association between EZH2, various survival measures, and clinicopathological features of various cancer types. We found that EZH2 was significantly associated with OS, DFS, MFS, PFS, CSS, and DSS, but not RFS, which was further confirmed by the pooled results of the multivariate analysis. This result suggested that EZH2 might be an independent prognostic factor for cancer survival. We found that EZH2 was associated with distant metastasis in esophageal carcinoma. EZH2 was associated with pT stage, N status, Fuhrman grade, and TNM stage in renal cell carcinoma. EZH2 was correlated with histological grade, estrogen receptor expression, and progesterone receptor expression in breast cancer. Further, we found that EZH2 was associated with pN stage and T status in gastric cancer, and with differentiation in NSCLC.

Significant heterogeneity was observed in most of our analyses. Subgroup analysis revealed that high EZH2 expression might predict poor OS in NSCLC, endometrial carcinoma, and oral tongue carcinoma, as well as poor DFS in renal cell carcinoma. However, these positive results were only based on a limited number of studies (two to four), and for the other types of cancer analyzed herein, either only one study was included or no significant association was detected. In a previous meta-analysis that included four Asian studies [63], it was observed that the OS of EZH2-negative patients was shorter than that of patients with positive expression for gastric cancer (HR = 0.54, 95% CI: 0.05–1.03), which was similar to our finding (HR = 0.88, 95% CI: 0.23–3.34). Furthermore, in another article concerning multiple prognostic biomarkers for NSCLC, CCNE1 and VEGF were the best two indicators of prognosis [64]. However, it is not clear how these two indicators compare with EZH2 because they were analyzed in meta-analyses, whereas EZH2 was analyzed in an individual study. In our study, however, we have focused on the role of EZH2 in multiple cancers (instead of different markers for a specific carcinoma). The current meta-analysis included three studies regarding the role of EZH2 in the prognosis of NSCLC. After pooling the data, we obtained results that were consistent with the above article. However, we cannot conclude that there is a definite association between EZH2 and a specific type of carcinoma, because of the limited number of studies that were available for any one type of cancer. Moreover, in a study of colorectal carcinoma, it was reported that the C/C allelic variant of EZH2 was more significantly correlated with poor PFS and OS than were two other variants (C/T or T/T)[65]. In future studies, we can additionally investigate the prognostic significance of EZH2 single-nucleotide polymorphisms for cancer survival.

In the current study, subgroup analyses revealed that patients with preoperative treatment presented with a smaller HR than those without preoperative treatment. This finding might imply a role for preoperative treatment as an interfering factor in the analysis of the association between EZH2 and cancer survival (due to its survival benefits). In light of this, we should consider preoperative treatment when applying EZH2 status to predict the OS of patients with cancer. Further investigations are warranted to verify our results, owing to the potential biases that could result from the limited number of studies with preoperative treatment, as well as the heterogeneity of surgery and preoperative treatment data.

Furthermore, we found that high EZH2 expression was more closely associated with poor OS in Asian patients than in Western populations, in which EZH2 had no significant predictive value for DFS. However, there were only two studies of DFS in Western patients, resulting in a loss of comparative strength for the pooled data, and inevitably introducing considerable bias. The results based on higher-quality reports revealed that high EZH2 expression was significantly associated with OS, but not with DFS or RFS. Moreover, sample size was found to be a significant source of heterogeneity, and HRs were smaller in large studies than in small studies. Therefore, we might have overestimated the significance of EZH2 in predicting cancer survival, as a consequence of the disproportionate contribution of results from low-quality or relatively small studies. Similarly, our analysis of EZH2’s associations with MFS, PFS, CSS, and DSS were based on only two or three studies, with inevitable publication bias. Accordingly, no firm conclusions can be drawn at present.

Meta-regression and sensitivity analyses did not alter the significant correlation of EZH2 with survival outcomes or reveal any significant sources of heterogeneity. However, certain stratifying covariates might contribute to the limited statistical power of meta-regression. Cumulative meta-analyses (by year) of did not reveal any obvious trends of change in the HRs of OS, DFS, or RFS over time. The analyses therefore indicated that the bias due to publication year was minor, despite one or two articles reporting results that deviated from the stable trend.

TNM stage, lymph node metastasis, histological type, and a number of other clinicopathological features are known to reflect the patient’s condition in terms of cancer prognosis. In our study, we also observed prognostic significance of EZH2 in multiple cancers. Thus, we speculate that there might be an association between EZH2 and some clinicopathological features of cancer. This hypothesis was confirmed in certain situations (mentioned above) without significant heterogeneity. However, in several cancer types, only a few clinicopathological features were correlated with EZH2. Nonetheless, our results did not necessarily indicate a significant association, because only a limited number of clinicopathological features were analyzed, and because few studies were included.

Our study’s results differ from those of a previous meta-analysis, which suggested that EZH2 expression was significantly associated with TNM stage (n = 9) and lymph node metastasis (n = 7), but not with T status (n = 5) in Asia[63]. These differences might have arisen from the different number of studies included. The relationship between EZH2 and clinicopathological features might have been shown more definitively by our study if there were more large-scale studies with consistent baselines (consistent baselines would have allowed us to combine the data more effectively). Thus, the prognostic significance of EZH2 in cancer might be explained in greater detail as additional data become available. Moreover, the independent roles of clinicopathological features were not analyzed in our study because the data were limited and inconsistent. This topic should be explored further. If certain independent clinicopathological features for cancer survival were identified, the combination of EZH2 status and these clinicopathological factors might provide more accurate assessments of cancer prognosis.

To the best of our knowledge, this is the first meta-analysis to evaluate the prognostic value of EZH2 in multiple cancers. There are several strengths to our meta-analysis. The included studies were conducted in 11 different countries, reducing the potential for bias due to racial, geographical, etiological, and economic-social factors, and thereby making our results more universally applicable. Moreover, a total of 49 studies were included with a sizable number of patients to compensate for the drawbacks of individual studies. The quality of the included reports was mostly adequate, with 60.4% having a quality score of ≥80%. Additionally, many factors that could potentially influence the pooled results were extracted to objectively assess the data. Subgroup, meta-regression, and sensitivity analyses were performed to address this issue.

However, the limitations of our study cannot be ignored. First, this study analyzed the prognostic significance of EZH2 in various types of carcinoma, rather than a single specific type. This resulted in considerable bias due to heterogeneous baselines of different cancer types. Second, cancer staging and EZH2 cut-off values were not consistent across studies, and the definitions of outcome measures were not provided in all reports. For example, the level of EZH2 expression was defined as positive or negative in some studies, and as high or low in other studies. Various cut-off values were employed. For our study, we defined EZH2 expression greater than the corresponding cut-off values as high. Other EZH2 expression was defined as low. This simple procedure might have introduced considerable heterogeneity. Third, surgery was not the only treatment for enrolled patients, and differences in other therapies could have increased the baseline heterogeneity. Fourth, some analyses were based on a limited number of studies, leading to inevitable bias in our analysis of the association between high EZH2 expression and OS. Moreover, we only included studies published in English, thus possibly missing results from studies reported in other languages, unpublished studies, and conference abstracts that might describe non-significant data. Therefore, our pooled results might be overestimates, owing to such reporting bias. However, a number of negative results were identified through by our study’s complete literature search, to minimize publication bias. Fifth, the data we employed did not include details for individual patients, and it was necessary to obtain eight estimates by calculation and 19 by survival curve reconstruction. Thus, considerable bias was inevitably introduced, owing to disparities with the original estimates. Finally, the accrual period of the included studies ranged from 1979 to 2012, which might have resulted in bias due to changes in detection techniques, improvements in surgical skills, and the evolution of medical treatments over time.

In conclusion, our results indicated that EZH2 might be an independent prognostic factor for OS, DFS, MFS, PFS, CSS, and DSS in multiple cancer types. It might therefore serve as a novel, broad biomarker of poor prognosis in various cancer types, potentially helping to identify high-risk patients and improve survival by allowing early therapeutic interventions. However, these suggestions should be further confirmed in large-scale prospective clinical studies.

## Supporting Information

S1 File Forest plots for the meta-analysis of the independent role of EZH2 expression in cancer survival in multiple cancers. The following cancer survival measures were analyzed: overall survival (Figure A in S1 File), disease-free survival (Figure B in S1 File), and recurrence-free survival (Figure C in S1 File). The segments represent the 95% confidence intervals (CIs) of each study. The diamond represents the overall effect size, and the diamond’s width represents the overall 95% CI.
(TIF)

S2 File Forest plots for meta-analyses of the association between EZH2 expression and cancer survival. The following cancer survival measures were analyzed: DFS (Figure A in S2 File) and RFS (Figure B in S2 File). The segments represent the 95% confidence intervals (CIs) of each study. The diamond represents the overall effect size, and the diamond’s width represents the overall 95% CI.
(TIF)

S3 File Funnel plot for the assessment of publication bias in our analysis of the correlation between EZH2 expression and overall survival in various cancer types. (TIF)

S1 Table Detailed characteristics of studies included in the meta-analysis. (DOC)

S2 Table Publication bias regarding the analysis of the association between EZH2 expression and OS, DFS, and RFS. (DOC)

S3 Table Summary of the excluded studies and the reasons for exclusion. (DOC)

S4 Table PRISMA 2009 checklist. (DOC)
