# Supplementary figures and images for: Enhancer of Zeste Homolog 2 as an Independent Prognostic Marker for Cancer: A Meta-Analysis
Source: PLoS One. 2015 May 14;10(5):e0125480. doi: 10.1371/journal.pone.0125480 (PMC4431777; doi:10.1371/journal.pone.0125480)

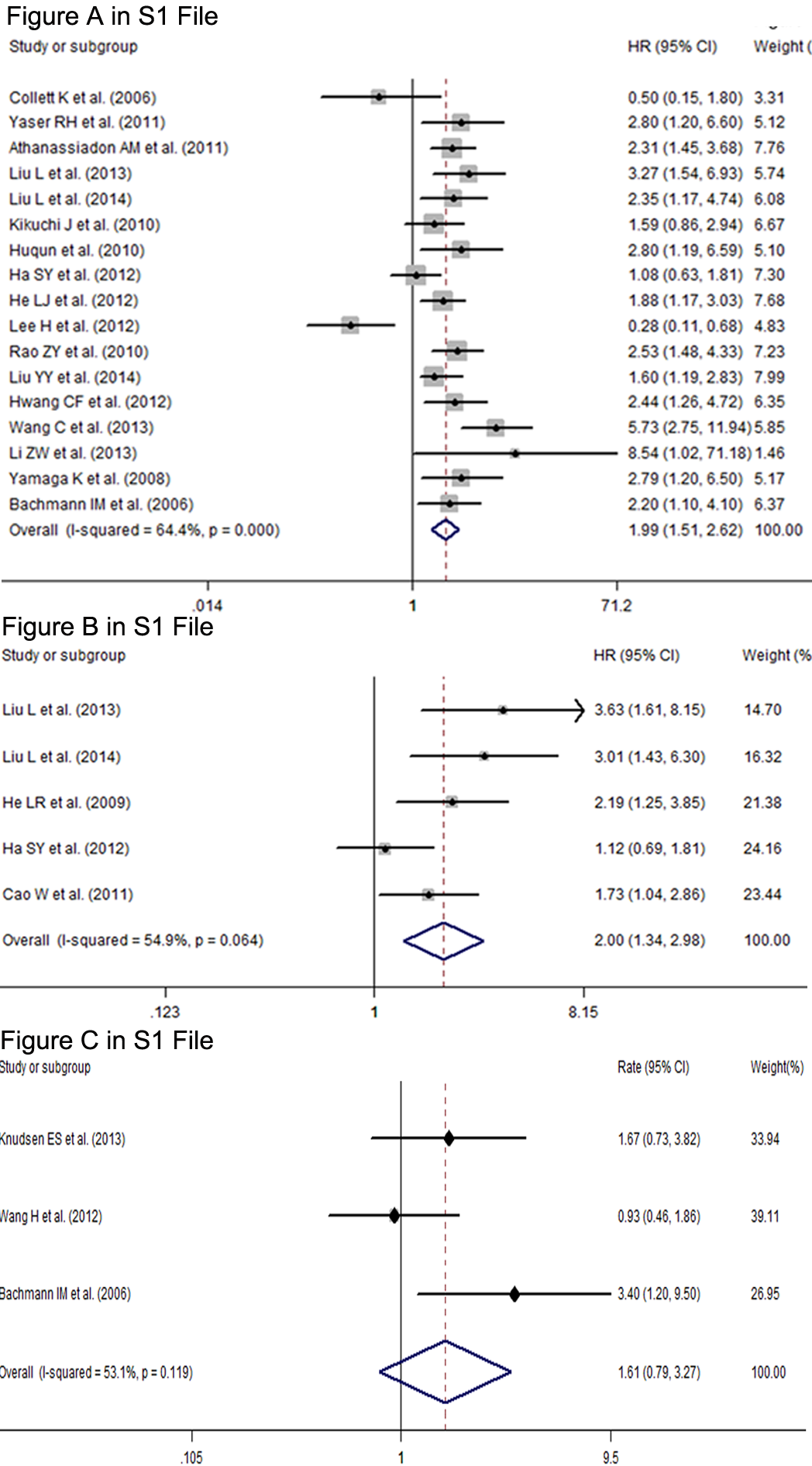

Supplement: S1 File — The following cancer survival measures were analyzed: overall survival (Figure A in S1 File), disease-free survival (Figure B in S1 File), and recurrence-free survival (Figure C in S1 File). The segments represent the 95% confidence intervals (CIs) of each study. The diamond represents the overall effect size, and the diamond’s width represents the overall 95% CI. (TIF) [file pone.0125480.s001.tif]

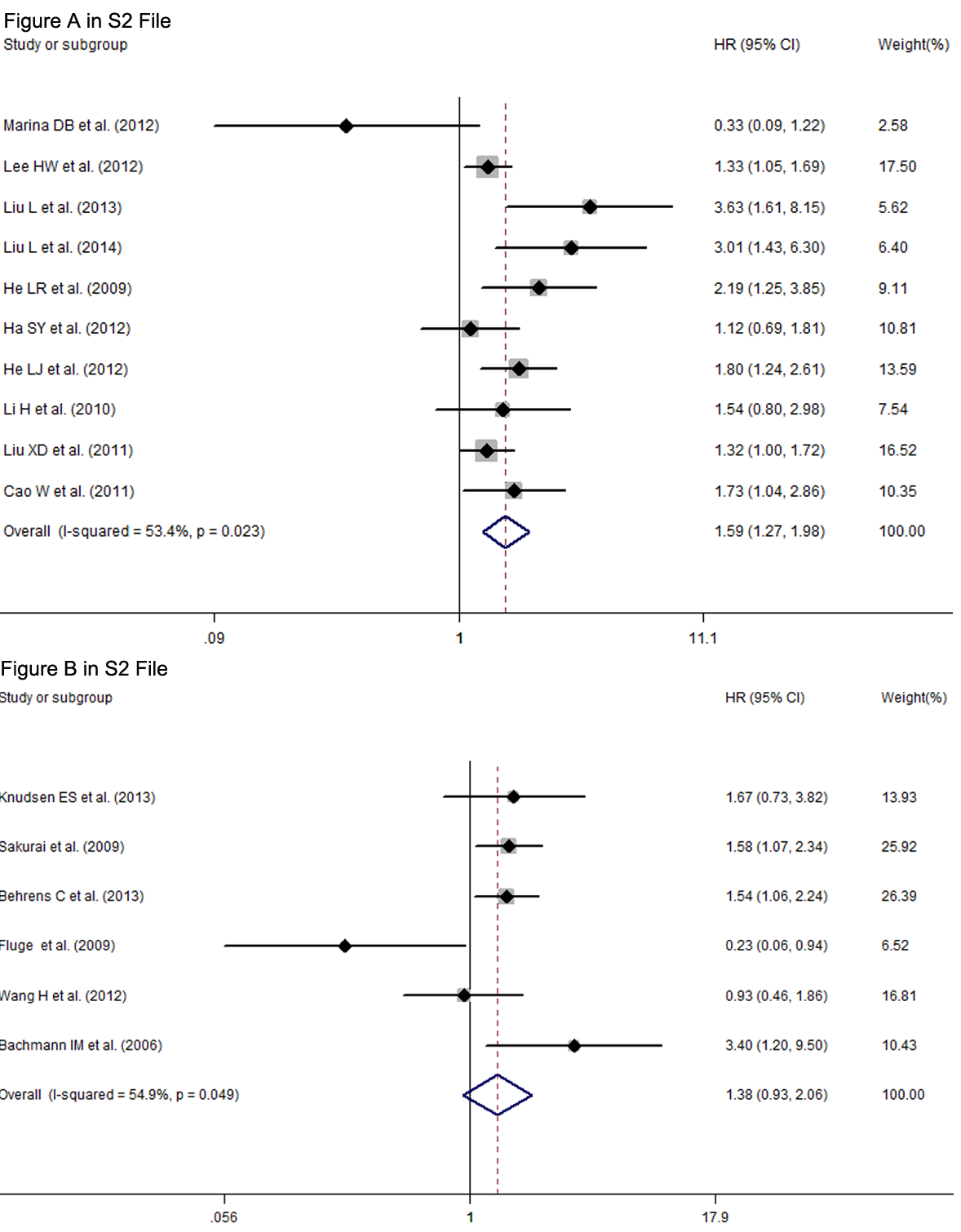

Supplement: S2 File — The following cancer survival measures were analyzed: DFS (Figure A in S2 File) and RFS (Figure B in S2 File). The segments represent the 95% confidence intervals (CIs) of each study. The diamond represents the overall effect size, and the diamond’s width represents the overall 95% CI. (TIF) [file pone.0125480.s002.tif]

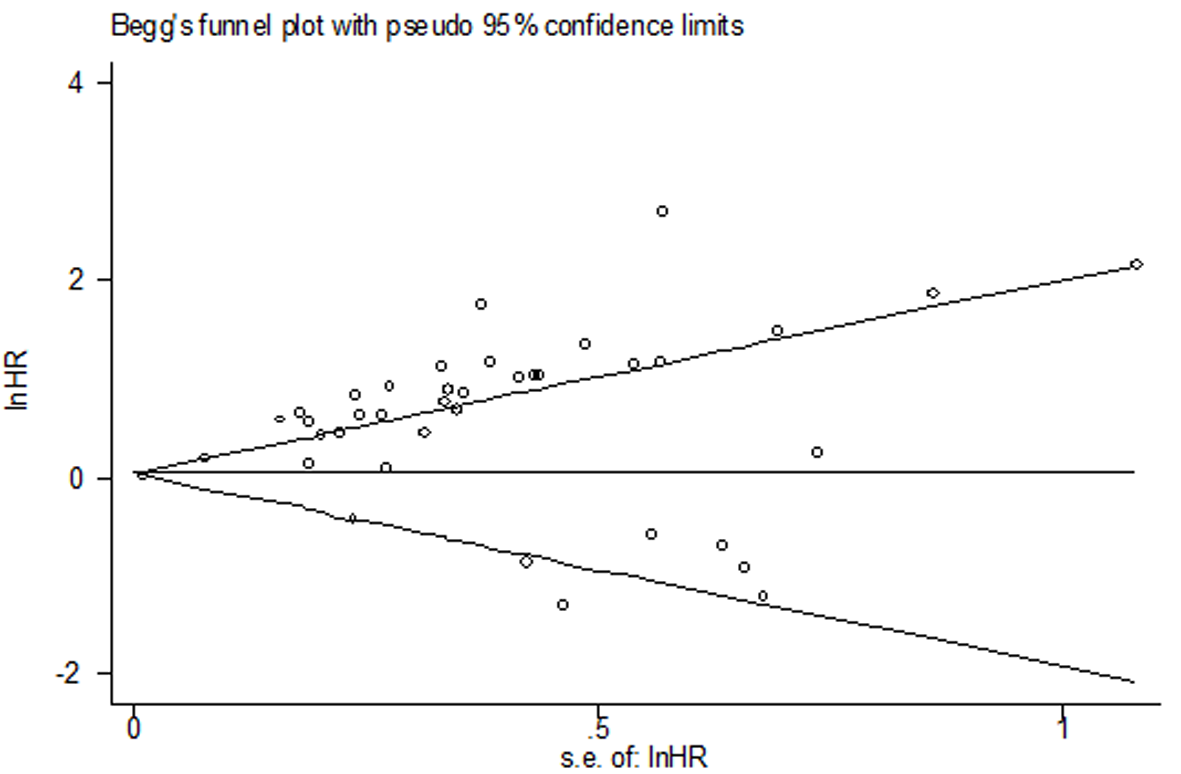

Supplement: S3 File — (TIF) [file pone.0125480.s003.tif]
