# Supplementary material for: Enhancer of Zeste Homolog 2 as an Independent Prognostic Marker for Cancer: A Meta-Analysis
Source: PLoS One. 2015 May 14;10(5):e0125480. doi: 10.1371/journal.pone.0125480 (PMC4431777; doi:10.1371/journal.pone.0125480)
Supplement: S1 Table — (DOC) [file pone.0125480.s004.doc]

**S1 Table. Detailed characteristics of studies included in the meta-analysis**

| **Study** | **Year** | **Region** | **Tumor type** | **Sample size (n)** | **Clinical stage of tumor** | **Cut-off value** | **Elevated EZH2 (%)** | **Treatment** | **Outcome measure** | **Definition of outcome measures** | **Survival analysis** | **Quality score**  **(%)** |
| --- | --- | --- | --- | --- | --- | --- | --- | --- | --- | --- | --- | --- |
| Collett K et al. | 2006 | Norway | Aggressive breast cancer | 190 | NA | High vs low: staining index of 3 | 47.4 | NA | OS | NA | Univariate and multivariate analysis | 85.9 |
| Yaser RH et al. | 2011 | USA | Triple negative breast cancer | 261 | 1-4 (AJCC TNM stage) | High vs low: staining index: 0,1 vs 2,3+ | 33.0 | Surgery, no preoperative treatment | OS | OS: from primary surgery to cancer-related death | Univariate and multivariate analysis | 88.8 |
| Gong Y et al. | 2011 | USA | Inflammat-ory breast cancer | 74 | NA | Positive vs negative: 10% of nuclear staining of invasive cancer cells | 75.7 | Mastectomy with or without axillary lymph node dissection, neoadjuvant chemotherapy or radiation therapy | OS and DSS | OS: from the date of initial pathologic diagnosis to the date of death from any cause or the last contact;  DSS: from the date of initial diagnosis to the disease-related death | Univariate and multivariate analysis | 84.6 |
| AthanassiadonAM et al. | 2011 | Greece | Breast cancer | 100 | Histological grade I-III (Scarff Bloom Richardson Classification) | Positivevs negative: 10% of positive tumor cells | 64.0 | Surgery without postoperative adjuvant therapy | OS | NA | Univariate and multivariate analysis | 78.8 |
| Marina DB et al. | 2012 | Brazil | Triple negative and basal like breast cancer | 140 | 1-4 (AJCC TNM stage) | High vs low: staining index: 1,2 vs 3,4 | 67.5 | NA | OS and DFS | NA | Univariate and multivariate analysis | 82.4 |
| Alford SH et al. | 2012 | USA | Familial early-stage breast cancer | 480 | Stage I and node positive Stage IIA | High vs low: staining index: 1,2 vs 3,4 | 42.5 | NA | MFS | NA | Univariate and multivariate analysis | 76.0 |
| Knudsen ES et al. | 2013 | USA | Ductal carcinoma in situ and invasive breast cancer | 236 | NA | High vs low: 15% of nuclear expression | 85.0 | Surgery,no preoperative treatment | RFS | RFS:from surgery to the relevant pathologic diagnosis | Univariate and multivariate analysis | 80.8 |
| Wagener N et al. | 2010 | Germany | Renal cell carcinoma | 520 | I-IV (Tumor node metastasis staging system of 2002) | High vs low: 25% of nuclear staining | 8.85 | Surgery, no adjuvant therapy | CSS | CSS: from the primary surgery to the end of follow-up or cancer-specific death | Univariate and multivariate analysis | 88.3 |
| Xu B et al. | 2012 | Canada | Clear cell renal cell carcinoma | 65 | T3-4 (AJCC TNM stage) | High vs low: H-score 0.5; H-score 10 | 36.4;48.8 | Surgery | OS | NA | Univariate and multivariate analysis | 80.7 |
| Sakurai T et al. | 2012 | USA | Renal cell carcinoma | 100 | NA | Positive vs negative: 50% of nuclear staining | 46 | Surgery | OS and RFS | NA | NA | 73.0 |
| Lee HW et al. | 2012 | Korea | Renal cell carcnioma | 171 | TNM 1-4 (the 7^th^ edition of AJCC) | High vs low: staining intensity score of positive tumor cells of 2 | 41.5 | Surgery | OS and DFS | OS: from the surgery to the last follow-up or death;  DFS: from the surgery to clinical relapse or the last follow-up without recurrence or metastasis | NA | 76.9 |
| Liu L et al. | 2013 | China | Renal cell carcinoma | 186 | TNM I-IV (AJCC TNM classification system) | High vs low: staining index of 2 | NA | Surgery without adjuvant therapy | OS and DFS | OS: from surgery to death from any cause;  DFS: from surgery to recurrence or metastasis | Univariate and multivariate analysis | 89.0 |
| Liu L et al. | 2014 | China | Renal cell carcinoma | 357 | TNM I-IV (AJCC TNM classification system) | High vs low: staining index of 2 | 33.3 | Surgery without adjuvant therapy | OS and DFS | OS: from surgery to death from any cause;  DFS: from surgery to recurrence or metastasis | Univariate and multivariate analysis | 78.5 |
| Hayashi A et al. | 2014 | Japan | Upper urinary tract carcinoma | 171 | TNM stage pTa, pTis, pT1-4 | Positive vs negative : 50% of positive cells | 55.0 | Surgery without neoadjuvant chemotherapy | MFS and CSS | NA | Univariate and multivariate analysis | 80.6 |
| Wang H et al. | 2012 | USA | Invasive urothelial carcinoma of the bladder | 81 | 1-4 (AJCC TNM staging system for bladder cancer) | High vs low: staining index of 2 | NA | Radical cystectomy without previous intravesical therapy | RFS | NA | Univariate and multivariate analysis | 84.5 |
| Varambally S et al. | 2002 | USA | Prostate cancer | 32 | NA | NA | NA | Surgery | Failure-free survival | From surgery to recurrence of cancer | Univariate and multivariate analysis | 85.9 |
| Laitinen S et al. | 2007 | Finland | Prostate cancer | 213 | Gleason score and pT-stage | High vs low: staining intensity of 50% | 6.57 | Prostatectomy and three patients undergone neoadjuvant and one adjuvant hormonal treatement | PFS | NA | Univariate and multivariate analysis | 83.7 |
| Kikuchi J et al. | 2010 | Japan | Non-small cell lung cancer | 154 | I-IV(AJCC guidelines for postoperative tumor lymph node-metastasis TNM classification) | High vs low: 25% of positive nuclear staining | 62.0 | Surgery | OS | NA | Univariate and multivariate analysis | 84.9 |
| Huqun et al. | 2010 | Japan | Non-small cell lung cancer | 106 | pTNM Stage I | High vs low: staining intensity of 50% | 50.0 | Surgery | OS | OS: from surgery to end of follow-up or death | Univariate and multivariate analysis | 82.8 |
| Behrens C et al. | 2013 | Canada | Non-small cell lung cancer | 320 | I-III(IASLC) | High vs low: an SI score of 41.7 | 50.0 | Surgery with or without adjuvant therapy | OS and RFS | OS: from surgery to death or last contact;  RFS: from surgery to recurrence or last contact | Univariate and multivariate analysis | 84.5 |
| He LR et al. | 2009 | China | Esophageal carcinoma | 98 | II-IV (the 6^th^  Edition of TNM classification of UICC 2002) | High vs low: staining intensity of 50% | 54.1 | Chemoradiotherapy without previous treatment | MFS,PFS and DRS | MFS/PFS/DDS: from the diagnosis to tumor metastasis, tumor progression and cancer-relative death | Univariate and multivariate analysis | 86.5 |
| Ymada A et al. | 2011 | Japan | Esophageal carcinoma | 136 | T1-4 (UICC TNM staging system) | Positive vs negative: 5% of positive cells | 14.0 | Surgery without preoperative therapy | DFS | NA | Univariate analysis | 79.4 |
| Ha SY et al. | 2012 | Korea | Esophageal carcinoma | 164 | NA | High vs low: an SI score of 10 | 52.4 | Curative surgical resection without preoperative treatment | OS and DFS | OS/DFS: from surgery to death or recurrence | Univariate and multivariate analysis | 84.5 |
| Wang HR et al. | 2013 | China | Esophageal carcinoma | 102 | TNM Stage I-IV | Positive vs negative: staining intensity of 50% | NA | Radial surgery without preoperative treatment | OS | NA | Univariate and multivariate analysis | 63.0 |
| Mimori K et al. | 2005 | Japan | Colorectal carcinoma | 61 | Dukes stage A-D | High vs low: T/N ratio:1.45 | 52.5 | Surgery | OS | NA | NA | 59.9 |
| Fluge Ø et al. | 2009 | Norway | Colorectal carcinoma | 290 | TNM stage II-III | High vs low: an SI score:0-3 vs 4-9 | 17.0 | Surgery or surgery with adjuvant therapy | RFS and CSS | NA | Univariate and multivariate analysis | 85.9 |
| Wang CG et al. | 2010 | China | Colorectal carcinoma | 119 | I-IV ( TNM classification) | Positive vs negative: 30% of positive cells in a section | 68.9 | Surgery without preoperative chemotherapy or radiotherapy | OS | NA | Univariate and multivariate analysis | 84.6 |
| Matsukawa Y et al. | 2005 | Japan | Gastric cancer | 83 | I-IV(the Japanese classification of Gastric Carcinoma) | High vs low: intense immunoreactivity of 50% | 56.6 | Surgery | CRS | CRS: death attributed only to cancer | NA | 72.4 |
| Choi JH et al. | 2010 | Korea | Gastric cancer | 137 | I-IV (AJCC classification) | Median staining index: 6.0 | 60.6 | Surgery with or without systematic chemotherapy or received oral 5-fluorouracil | OS | NA | NA | 66.9 |
| He LJ et al. | 2012 | China | Gastric cancer | 117 | I-IV (AJCC/International Union against Cancer tumor-node-metastasis TNM classification system) | High vs low: 50% of tumor cells in the triplicate tissue spots on TMA sections | 70.1 | Surgery | OS and DFS | NA | Univariate and multivariate analysis | 78.8 |
| Lee H et al. | 2012 | Korea | Gastric cancer | 178 | The 7^th^ AJCC/ UICC TNM classification system | High vs low: staining score: 3 | 92.1 | Surgery with adjuvant chemotherapy | OS | NA | Univariate and multivariate analysis | 78.8 |
| Yonemitsu Y et al. | 2009 | Japan | Hepatocellular carcinoma | 86 | TNM I-IVa | Positive vs negative :NA | 66.3 | Surgery | OS and RFS | NA | Univariate and multivariate analysis | 61.7 |
| Nakagawa S et al. | 2013 | Japan | Cholangiocarcinoma | 86 | I-IV (the International Union against Cancer tumor-node-metastasis classification) | High vs low: 10% of positively –stained cells | ICC 55.6;  ECC 41.5 | Surgery, chemotherapy | OS | OS: from surgery to death from any cause or the last follow-up | Univariate and multivariate analysis | 85.5 |
| Liu DC et al. | 2010 | China | Gallbladder adenocarcinoma | 67 | T1-4 (Standard criteria of T stage) | Positive vs negative: 25% of positive cells | 53.7 | Surgery | OS | NA | Multivariate analysis | 81.0 |
| Rao ZY et al. | 2010 | China | Ovarian carcinoma | 179 | I-IV (FIGO stage) | High vs low: 50% of cells with positive immunoreactivity in nuclei | 49.7 | Surgery, preoperative radiation or chemotherapy | OS | NA | Univariate and multivariate analysis | 80.6 |
| Li H et al. | 2010 | USA | Ovarian carcinoma | 134 | Tumor stage 1-4, underterminded | High vs low: H-score:100 | 65.7 | Surgery | OS and DFS | OS: from diagnosis to death from any cause or the last follow-up;  DFS: from surgery to the first recurrence | NA | 61.4 |
| Liu YY et al. | 2014 | China | Cervical cancer | 101 | Internation Federation of Gynecology and Obstetrics criteria | Positive vs negative: staining index of 4 | 68.3 | surgery | OS | From surgery to death or end of follow-up | Univariate and multivariate analysis | 84.9 |
| Zhou J et al. | 2012 | USA | High grade endometrial carcinoma | 202 | 2009 International Federation of Gynecology and Obstetrics criteria | High vs low: an staining index of 2 | Type-1 tumors: 7.6%;  Type-2 tumors 63% | Surgery without preoperative treatment | OS | NA | Univariate and multivariate analysis | 65.7 |
| Liu XD et al. | 2011 | China | Nasopharyngeal carcinoma | 47 | TNM staging II-IV (UICC 2003) | High vs low: median positive staining index of 72.9% | 72.3 | Radiotherapy | DFS | NA | NA | 59.9 |
| Hwang CF et al. | 2012 | China | Nasopharyngeal carcinoma | 89 | Stage I-IV (AJCC 2002 on cancer) | High vs low: nuclear labeling index of 60% | 47.2 | Radiotherapy | OS and DSS | From the date of RT to the date of event | Univariate and multivariate analysis | 80.6 |
| Wang C et al. | 2013 | China | Oral tongue squamous cell carcinoma | 67 | TNM system by UICC | High vs low: an IS of 2 | 34.3 | Surgery without preoperative adjuvant therapy | OS | NA | Univariate and multivariate analysis | 83.2 |
| Kidani K et al. | 2008 | Japan | oral  squamous cell carcinomas | 83 | II-IV (UICC tumor size nodal metastasis distant metastasis TNM classification) | Positive vs negative: an LI of 50% | 50.6 | Surgery | OS | NA | NA | 66.0 |
| Li ZW et al. | 2013 | China | Tongue cancer | 84 | Clinical stage I-IV | High vs low: immunoreactive score of 4 | 64.3 | Surgery without chemotherapy or radiotherapy | OS | NA | Univariate and multivariate analysis | 77.2 |
| Cao W et al. | 2011 | China | Head and neck squamous cell carcinoma | 117 | III-IVA (UICC tumor-node-metastasis TNM classification system) | High vs low: an LI score of 135 | 50.4 | Surgery without preoperative treatment | OS and DFS | NA | Univariate and multivariate analysis | 92.2 |
| Yamaga K et al. | 2008 | Japan | Soft tissue carcinoma | 104 | Histological grade 1-3 (criteria of the FNCLCC/French system) | High vs low: staining index of 10.8% | NA | Surgery with preoperative treatment | OS | NA | Univariate and multivariate analysis | 86.5 |
| ChangChien YC et al. | 2012 | Hungary | Synovial sarcoma | 55 | WHO classification | High vs low: total score of 4 | NA | Surgery without preoperative treatment | OS | NA | Univariate analysis | 72.2 |
| Vekony H et al. | 2008 | The Netherlands | Salivary gland adenoid cystic carcinoma | 21 | NA | High vs low: percentage of tumor cells with positive staining-25% | 28.6 | Surgery with or without postoperative radiotherapy | OS, PFS | NA | Univariate analysis | 85.0 |
| Bachmann IM et al. | 2006 | Norway | 1-Nodular melanoma,  2-Prostate cancer,  3-Endometrial carcinoma, | 696 | 1-2: TNM category;  3:FIGO stage;  4:histologic grade | High vs low: 1,4:SI=3  2: SI=4  3: SI=6 | 1:28.2;  2: 8.65;  3:15.5;  4: 47.4 | Surgery | OS and RFS | NA | Univariate and multivariate analysis | 84.9 |
| Takawa M et al. | 2011 | Japan | 1. NSCLC, 2. Colorectal carcinoma, | 464 | 1. pT1–pT3, pN0–pN2,and pM0 (Pathologic TNM classification of the UICC); 2. WHO classification 3. NA | Positive vs negative :brown staining appreciable in the nucleus | 1: 46.2;  2:91.4;  3:NA | Surgery with adjuvant therapy but without preoperative treatment | OS | OS: from surgery to death from any cause or the last follow-up | Univariate and multivariate analysis | 84.5 |

SI(staining index score) or LI(labeling index): staining intensity*proportion of positive stained cells. Total score: nuclear staining intensity and the percentage of tumor cells with positive nuclear reaction were summed.

OS: overall survival; DFS: disease-free survival; RFS: recurrence-free survival; MFS: metastasis-free survival; CSS: cancer-specific survival; DSS: disease-specific survival.

Locally advanced stage T3 and above: H-score 0.5; metastatic renal cell carcinoma: H-score 10; H-score is defined as the product of the percentage of tumor cells showing EZH2 labeling (0–100) multiplied by the labeling intensity (0–3).

AJCC: the American Joint Committee on Cancer.

UICC: the International Union Against Cancer.

IASLC: The world health organization and International Association for the study of lung cancer classification.

ICC: intrahepatic cholangiocarcinoma; ECC: extrahepatic cholangiocarcinoma.

Publication bias regarding the analysis of the association between EZH2 expression and OS, DFS and RFS.

|  |  | |  | |  |
| --- | --- | --- | --- | --- | --- |
|  |  |  |  |
|  |  |  |  |  |  |
|  |  |  |  |  |  |
|  |  |  |  |  |  |
|  |  |  |  |  |  |
|  |  |  |  |  |  |
|  |  |  |  |  |  |
|  |  |  |  |  |  |
|  |  |  |  |  |  |
|  |  |  |  |  |  |
|  |  |  |  |  |  |
|  |  |  |  |  |  |

|  |  |
| --- | --- |
|  |  |
|  |  |
|  |  |
|  |  |
|  |  |
|  |  |
|  |  |
|  |  |
|  |  |
|  |  |
|  |  |
|  |  |
|  |  |
