# Supplementary material for: Enhancer of Zeste Homolog 2 as an Independent Prognostic Marker for Cancer: A Meta-Analysis
Source: PLoS One. 2015 May 14;10(5):e0125480. doi: 10.1371/journal.pone.0125480 (PMC4431777; doi:10.1371/journal.pone.0125480)
Supplement: S2 Table — (DOC) [file pone.0125480.s005.doc]

**S2 Table.** Publication bias regarding the analysis of the association between EZH2 expression and OS, DFS and RFS.

| **Outcome measures** | **Egger’s test** | | **Begg’s or Begg-Mazumdar test** | | **Presence of publication bias** |
| --- | --- | --- | --- | --- | --- |
|  | **t** | **p value** | **z (continuity corrected)** | **p value (continuity corrected)** |  |
| **OS** | 5.48 | 0.00 | 1.41 | 0.16 | Significant |
| **Region: Asian countries** | 1.58 | 0.13 | 1.90 | 0.06 | Non-significant |
| **Region: Western countries** | 2.92 | 0.01 | 1.03 | 0.30 | Significant |
| **Sample size <150** | 8.35 | 0.00 | 0.51 | 0.61 | Significant |
| **Sample size ≥150** | -0.10 | 0.92 | 0.77 | 0.44 | Non-significant |
| **Surgery without preoperative treatment** | 2.25 | 0.04 | 2.38 | 0.02 | Significant |
| **Surgery with preoperative treatment** | -0.22 | 0.84 | — | — | Non-significant |
| **Quality score <83.0** | 3.09 | 0.01 | 1.36 | 0.17 | Significant |
| **Quality score ≥ 83.0** | 2.64 | 0.02 | 1.54 | 0.12 | Significant |
| **DFS** | 0.82 | 0.44 | — | — | Non-significant |
| **RFS** | -0.78 | 0.48 | — | — | Non-significant |

OS: overall survival; DFS: disease-free survival; RFS: recurrence-free survival.
