# Supplementary material for: Enhancer of Zeste Homolog 2 as an Independent Prognostic Marker for Cancer: A Meta-Analysis
Source: PLoS One. 2015 May 14;10(5):e0125480. doi: 10.1371/journal.pone.0125480 (PMC4431777; doi:10.1371/journal.pone.0125480)
Supplement: S3 Table — (DOC) [file pone.0125480.s006.doc]

| **S3 Table.** Summary of the excluded studies and the reasons for exclusion. |  |
| --- | --- |
| **Articles** | **Reasons for exclusion** |
| 1. Mimori K, Ogawa K, Okamoto M, et al. Clinical significance of enhancer of zeste homolog 2 expression in colorectal cancer cases. EJSO 31, 376–380. | EZH2 expression (mRNA) with methods other than IHC |
| 2. Sudo T, Utsunomiya T, Mimori K, et al. Clinicopathological significance of EZH2 mRNA expression in patients with hepatocellular carcinoma. British Journal of Cancer 92, 1754 – 1758. | EZH2 expression (mRNA) with methods other than IHC |
| 3. Jansen M. P. H. M, Reijm E. A, Sieuwerts A. M, et al. High miR-26a and low CDC2 levels associate with decreased EZH2 expression and with favorable outcome on tamoxifen in metastatic breast cancer. Breast Cancer Res Treat 133:937–947. | EZH2 expression (mRNA) with methods other than IHC |
| 4. Pietersen AM, Horlings HM, Hauptmann M, et al. EZH2 and BMI1 inversely correlate with prognosis and TP53 mutation in breast cancer. Breast Cancer Research 10(6): R109. | EZH2 expression (mRNA) with methods other than IHC |
| 5. Reijm E. A., Jansen M. P. H. M., Ritstier KR, et al. Decreased expression of EZH2 is associated with upregulation of ER and favorable outcome to tamoxifen in advanced breast cancer. Breast Cancer Res Treat 125:387–394. | EZH2 expression (mRNA) with methods other than IHC |
| 6. Cao W, Ribeiro R de O, Liu D, et al. EZH2 promotes malignant behaviors via cell cycle dysregulation and its mRNA level associates with prognosis of patient with non-small cell lung cancer. PLoS One 7(12):e52984. | EZH2 expression (mRNA) with methods other than IHC |
| 7. Xu CH, Hou ZB, Zhan P, et al. EZH2 regulates cancer cell migration through repressing TIMP-3 in non-small cell lung cancer. Med Oncol 30:713. | No sufficient data to calculate or approximate HR estimates |
| 8. Cao W, Younis RH, Li J, et al. EZH2 Promotes malignant phenotypes and is a predictor of oral cancer development in patients with oral leukoplakia. Cancer Prev Res 4:1816-1824. | No sufficient data to calculate or approximate HR estimates |
| 9. Lu H, Sun J, Wang F, et al. Enhancer of zeste homolog 2 activates wnt signaling through downregulating CXXC finger protein 4. Cell Death and Disease 4, e776. | No sufficient data to calculate or approximate HR estimates |
| 10. Eskander RN, Ji T, Huynh TB,et al. Inhibition of enhancer of zeste homolog 2 (EZH2) expression is associated with decreased tumor cell proliferation, migration, and invasion in endometrial cancer cell lines. Int J Gynecol Cancer;23: 997-1005. | No sufficient data to calculate or approximate HR estimates |
| 11. Pang J, Toy KA, Griffith KA, et al. Invasive breast carcinomas in Ghana: high frequency of high grade, basal-like histology and high EZH2 expression. Breast Cancer Res Treat 135:59–66. | No sufficient data to calculate or approximate HR estimates |
| 12. Gyorffy B, Surowiak P, Budczies J, et al. Online survival analysis software to assess the prognostic value of biomarkers using transcriptomic data in non-small-cell lung cancer. PLoS One 8(12): e82241. | No sufficient data to calculate or approximate HR estimates |
